# Prospective Voice Assessment After Thyroidectomy Without Recurrent Laryngeal Nerve Injury

**DOI:** 10.3390/diagnostics15010037

**Published:** 2024-12-27

**Authors:** Ivana Šimić Prgomet, Stjepan Frkanec, Ika Gugić Radojković, Drago Prgomet

**Affiliations:** 1 Phoniatric Center, Department of ENT and Head and Neck Surgery, University Hospital Center Zagreb, 10000 Zagreb, Croatia; simic.ivana24@gmail.com; 2 Department of ENT and Head and Neck Surgery, University Hospital Center Zagreb, 10000 Zagreb, Croatia; ikagugic@gmail.com (I.G.R.); dprgomet2@gmail.com (D.P.); 3 School of Medicine, University of Zagreb, 10000 Zagreb, Croatia

**Keywords:** voice disorders, thyroidectomy, RNL preservation

## Abstract

**Background**: Thyroidectomy, a surgical procedure for thyroid disorders, is associated with postoperative voice changes, even in cases without recurrent laryngeal nerve (RLN) injury. Our study evaluates the prevalence and predictors of voice disorders in thyroidectomy patients without RLN injury. **Methods**: Our single-center prospective study at the University Hospital Center Zagreb included 243 patients, with pre- and postoperative voice evaluations using acoustic analysis and videostroboscopy. Logistic regression, chi-square, MANOVA, and non-parametric tests assessed the impact of surgical, sociodemographic, and lifestyle factors. **Results**: The study analyzed 243 participants (141 lobectomy, 102 total thyroidectomy). Postoperative voice disorders occurred in 200 patients (100 lobectomy, 100 total thyroidectomy); 43 (17.7%) experienced no voice disorders. Significant associations were observed for surgery type (χ^2^ = 29.88, *p* < 0.001), with total thyroidectomy having higher risk, surgery duration (χ^2^ = 16.40, *p* < 0.001), thyroid volume (χ^2^ = 4.24, *p* = 0.045), and BMI (χ^2^ = 8.97, *p* = 0.011). Gender and age showed no significant correlation. Acoustic parameters differed significantly, with lobectomy patients showing better intensity, jitter, and shimmer values across postoperative measurements. Logistic regression identified surgery type (Exp(B) = 16.533, *p* = 0.001) and thyroid volume (Exp(B) = 2.335, *p* = 0.023) as predictors of voice disorders, achieving 82.7% classification accuracy. Multivariate analysis confirmed gender and surgery duration as significant contributors. Surgery duration exceeding 90 min and enlarged thyroid volume negatively influenced outcomes. Significant acoustic differences were also linked to BMI categories, with obese participants exhibiting poorer parameters, particularly shimmer and jitter. **Conclusions**: Surgery type, thyroid volume, BMI, and surgery duration are most likely significant predictors of postoperative voice disorders.

## 1. Introduction

The thyroidectomy, the surgical removal of the thyroid gland, is a well-established treatment for various thyroid disorders, including thyroid cancer, benign nodules, and hyperthyroidism [1]. While modern surgical techniques and intraoperative nerve monitoring have reduced the risk of recurrent laryngeal nerve (RLN) injury, patients often experience voice changes even in the absence of RLN damage [2]. These alterations can significantly affect quality of life, particularly in patients whose professions rely heavily on vocal performance [3,4]. Studies indicate that as many as 80% of patients may experience transient voice changes post-thyroidectomy, with some reporting persistent dysphonia [3,5]. Several mechanisms are hypothesized to explain voice changes in patients without RLN injury. One key factor is injury to the external branch of the superior laryngeal nerve (EBSLN), which innervates the cricothyroid muscle responsible for pitch control and projection [5,6]. Additionally, structural alterations from strap muscle dissection or laryngeal manipulation during surgery may impact vocal function by modifying the laryngeal framework [7,8,9]. Another potential contributor is intubation trauma; endotracheal intubation can cause mucosal injury or edema, further impacting postoperative vocal quality [10,11]. A multidimensional approach to assessing voice outcomes post-thyroidectomy is essential, combining acoustic analysis, perceptual evaluation, and patient-reported measures. Acoustic parameters such as jitter, shimmer, and harmonic-to-noise ratio provide objective data on vocal fold function, while tools like the Voice Handicap Index (VHI) allow for self-reported assessment of voice impact [3,12]. Current literature lacks comprehensive, prospective studies specifically focused on voice outcomes in patients without RLN injury. Existing studies are often retrospective, limited in sample size, or lack standardized assessment methodologies, reducing the generalizability of findings [13,14]. Some of these studies are presented in Table 1 [7,14,15,16,17,18,19]. Clarifying the prevalence, progression, and factors contributing to these voice changes is crucial to enhancing patient counseling, surgical techniques, and targeted rehabilitation. This study seeks to bridge these gaps by conducting a prospective analysis of voice changes in thyroidectomy patients without RLN injury. Using a multidimensional assessment approach, we aim to document the prevalence, nature, and progression of these voice alterations. Understanding these dynamics will provide a basis for improved surgical strategies and postoperative care, ultimately enhancing patient quality of life following thyroid surgery.

## 2. Materials and Methods

This single-center prospective study was conducted at the Department of ENT and Head and Neck Surgery, University Hospital Center Zagreb, Croatia. Eligible participants were patients aged 18 years or older who provided informed consent. Exclusion criteria included preoperative voice disorders of any cause, prior neck surgeries or irradiation, and recurrent laryngeal nerve (RLN) damage during thyroidectomy. The sample included 243 patients; initially there were 251 participants, but 8 were excluded due to RLN palsy (3 after partial lobectomy and 5 following total thyroidectomy). Data collection continued until 100 patients who had undergone total thyroidectomy and 100 patients who had undergone lobectomy and developed a voice disorder were recruited. Additionally, data were gathered from participants who did not develop a voice disorder postoperatively, comprising 43 individuals (17.7% of the total sample). Participants who developed a voice disorder after surgery were monitored through three postoperative assessments: 7 to 10 days after surgery, 3 months after surgery, and 6 months after surgery. All participants, regardless of recovery status, were followed until the end of the study as part of regular follow-up visits at the University Hospital Center Zagreb. During the evaluation period, participants did not undergo voice therapy. All surgeries were performed by a single experienced surgeon using a standard open technique, ensuring preservation of the infrahyoid musculature and RLN.

### 2.1. Objective Analysis of Voice Disorder (Acoustic Analysis)

Any deviation in the parameters of acoustic analysis was regarded as indicative of a voice disorder. Objective voice evaluation included selected acoustic and aerodynamic measures. Voice assessment involved sustained vocalization of the vowel /a/, recorded in an acoustic lab using a digital recorder and microphone. The Ling Waves SLP Suite Pro VPR program (WEVOSYS medical technology GmbH, Germany) analyzed these recordings. During recording, the microphone was positioned 30 centimeters from the subject’s mouth at a 45° angle. The recorded signal was converted from analog to digital using Ling Waves software 3.2. Physiological values were defined as follows, based on established literature:

- Fundamental frequency (Hz): Female: 206 Hz, Male: 120 Hz, Children: 300 Hz [20].
- Intensity (dB): Female: 68–74 dB, Male: 68–76 dB [21].
- Jitter (%): Normal range: 0 to 0.5% [22].
- Shimmer (%): Normal range: 0 to 5% [22].
- Maximum phonation duration (s): Female: 15–25 s, Male: 25–35 s [22].

### 2.2. Videostroboscopy

Digital videostroboscopy, the gold standard in diagnosing vocal cord pathology, was utilized based on Talbot’s law, with stroboscopic flashes synchronized to the vocal cord’s high-frequency vibrations. A symmetrical, periodic mucosal wave with complete vocal cord closure was considered normal. All participants underwent pre- and post-thyroidectomy video-stroboscopic examination, and patients with irregular findings due to RLN damage were excluded. During videostroboscopy, patients performed a series of vocal tasks to assess voice function. These included sustained phonation of vowels “aa”, “ee”, and “ii” at a comfortable pitch and volume. Loudness variation, from soft to loud and vice versa, was also tested. High-pitched phonation, often in falsetto, and low-pitched phonation were performed to evaluate vocal range. Connected speech tasks, such as reading a passage or speaking continuously, were included alongside coughing and throat clearing to observe glottic closure. Inspiratory phonation was tested to observe vocal fold behavior during inhalation. These tasks collectively provided a detailed evaluation of voice quality and function after thyroidectomy.

### 2.3. Statistical Analysis

The number of participants who developed the disorder, depending on the observed factors, was presented as percentages. A chi-square test was employed to examine the development of voice disorders after thyroidectomy based on surgical characteristics (type and duration of the surgery), preoperative thyroid volume, socio-demographic features (age, gender), and lifestyle indicators (smoking, alcohol consumption, and BMI). To assess the predictive potential of factors found to be significant, as well as the individual contribution of each factor to the development of voice disorders after thyroidectomy, logistic regression analysis was performed.

After presenting the descriptive indicators of voice parameters, it was determined that some distributions exhibited increased skewness or kurtosis. Consequently, differences in mean values of voice parameters based on surgical characteristics, thyroid volume, socio-demographic features, and lifestyle factors were tested using non-parametric methods. For variables with more than two participant groups (e.g., BMI), the Kruskal-Wallis test was used, whereas the Mann-Whitney U test was applied for variables with two participant groups (all other characteristics).

To examine the effect size of each factor on the linear combination of voice parameter values, a multivariate analysis of variance (MANOVA) was conducted. Finally, Pearson correlations between each voice parameter and the tested characteristics were calculated for all three measurements.

The analysis was performed using the SPSS 21 software package.

A *p*-value of <0.05 was considered statistically significant.

## 3. Results

### 3.1. Sample Characteristics

The study included a total of 243 participants, with 141 undergoing lobectomy and 102 undergoing total thyroidectomy. Among these, 200 participants developed a postoperative voice disorder, with an equal distribution between those who underwent lobectomy (*n* = 100) and those who underwent total thyroidectomy (*n* = 100). Conversely, 43 participants (17.7% of the sample) did not experience any postoperative voice disorder, with a breakdown of 41 in the lobectomy group and 2 in the total thyroidectomy group.

### 3.2. Surgical Characteristics and Socio-Demographic Data

In relation to surgery duration, 58 participants had operations lasting up to 60 min (56 lobectomies and 2 total thyroidectomies), 84 participants had operations between 61 and 90 min (65 lobectomies and 19 total thyroidectomies), 46 had durations between 91 and 120 min (17 lobectomies and 29 total thyroidectomies), and 55 participants had operations exceeding 120 min (3 lobectomies and 52 total thyroidectomies).

Regarding thyroid volume, 118 participants presented with a normal thyroid volume (68 lobectomies and 50 total thyroidectomies), while 125 participants had an enlarged thyroid (73 lobectomies and 52 total thyroidectomies). In terms of gender distribution, 42 participants were male (23 lobectomies and 19 total thyroidectomies), and 201 were female (118 lobectomies and 83 total thyroidectomies).

Participants’ ages were distributed as follows: 59 participants were aged between 20 and 39 years (31 lobectomies and 28 total thyroidectomies), 138 participants were between 40 and 59 years (80 lobectomies and 58 total thyroidectomies), and 46 were between 60 and 79 years (30 lobectomies and 16 total thyroidectomies).

For body mass index (BMI), 39 participants had a normal BMI (31 lobectomies and 8 total thyroidectomies), 111 participants were overweight (62 lobectomies and 49 total thyroidectomies), and 93 participants were classified as obese (48 lobectomies and 45 total thyroidectomies). (More detailed values are presented in Table 2).

### 3.3. Development of Voice Disorders Relative to Surgery Type, Socio-Demographic Characteristics, and Lifestyle Factors

A significant association was observed between the type of surgery and the development of voice disorders (χ^2^ = 29.88, *p* < 0.001), with 95.3% of those who did not develop a voice disorder having undergone lobectomy, compared to only 50% of those who developed a voice disorder.

Surgery duration was also significantly related to voice disorder development (χ^2^ = 16.40, *p* < 0.001). Among those whose surgery lasted 90 min or less, 86.0% did not develop a voice disorder, while 14.0% of those with surgery exceeding 90 min did not develop a voice disorder.

Thyroid volume showed a significant association with voice disorders (χ^2^ = 4.24, *p* = 0.045), with 62.8% of participants with normal thyroid volume not developing a voice disorder, compared to 37.2% of those with increased thyroid volume.

No significant association was found between gender and the development of voice disorders (χ^2^ = 0.41, *p* = 0.659), nor between age groups (χ^2^ = 0.14, *p* = 0.932).

A significant association was observed between BMI and voice disorder development (χ^2^ = 8.97, *p* = 0.011). Within the normal BMI group, 30.2% did not develop a voice disorder, compared to 23.3% of those classified as obese. Smoking status and alcohol consumption did not show statistically significant results. (More detailed values are presented in Table 3).

### 3.4. Acoustic Parameter Measurements and Differences Based on Surgery Type

At the first postoperative measurement (POM), significant differences were found between lobectomy and total thyroidectomy groups in several acoustic parameters. The mean intensity was 69.72 dB for lobectomy and 66.03 dB for total thyroidectomy (Mann-Whitney U = 6.54, *p* < 0.001). Mean jitter was 0.94% for lobectomy and 1.10% for total thyroidectomy (Mann-Whitney U = 3.55, *p* < 0.001), and shimmer values were 15.08% for lobectomy and 16.65% for total thyroidectomy (Mann-Whitney U = 3.87, *p* = 0.006). Mean phonation time was 17.48 s for lobectomy and 14.83 s for total thyroidectomy (Mann-Whitney U = 6.93, *p* < 0.001).

At the second POM, mean intensity was significantly higher in lobectomy patients (70.45 dB) compared to total thyroidectomy patients (67.84 dB) (Mann-Whitney U = 6.41, *p* = 0.001). Mean jitter was 0.57% for lobectomy and 0.73% for total thyroidectomy (Mann-Whitney U = 6.52, *p* < 0.001), while shimmer was 8.10% for lobectomy and 10.60% for total thyroidectomy (Mann-Whitney U = 6.39, *p* = 0.001).

At the third POM, lobectomy patients had a mean intensity of 70.58 dB, compared to 68.66 dB for total thyroidectomy patients (Mann-Whitney U = 6.34, *p* = 0.001). The mean jitter value was 0.49% for lobectomy and 0.59% for total thyroidectomy (Mann-Whitney U = 5.82, *p* = 0.046), and the mean shimmer was 6.16% for lobectomy and 7.62% for total thyroidectomy (Mann-Whitney U = 5.84, *p* = 0.041). FO at first POM and FO and phonation time at second POM and third POM did not show statistically significant results. (More detailed values are presented in Table 4).

### 3.5. Acoustic Parameter Differences Based on Surgery Duration

When comparing acoustic parameters based on surgery duration, significant differences were found at various postoperative measurements. At the first POM, intensity was 69.63 dB in surgeries up to 90 min and 65.93 dB in surgeries longer than 90 min (Mann-Whitney U = 3.78, *p* = 0.003). Jitter was 0.94% in surgeries up to 90 min and 1.11% in longer surgeries (Mann-Whitney U = 6.33, *p* = 0.001), and phonation time was 16.84 s in surgeries up to 90 min compared to 15.40 s in longer surgeries (Mann-Whitney U = 3.99, *p* = 0.014).

At the second POM, mean frequency (F0) was significantly higher in surgeries up to 90 min (180.80 Hz) than in surgeries exceeding 90 min (172.31 Hz) (Mann-Whitney U = 4.09, *p* = 0.028). Additionally, jitter and shimmer values were higher in surgeries over 90 min across both the second and third POMs (more detailed values are presented in Table 5.

### 3.6. Acoustic Parameter Differences Based on Thyroid Volume

Statistically significant results were obtained in the third POM for jitter values; 91 patients had normal thyroid volume, and 109 had enlarged thyroid gland. The mean values were 0.51 vs. 0.57, *p* = 0.49. No significant results were observed for other values. (More detailed values are presented in Table 6).

### 3.7. Acoustic Parameter Differences Based on Gender When Compared to Preoperative Measurement

Statistically significant results were found between genders in values of change in fundamental frequency and change in time of phonation across all three POMs, *p* < 0.001. There were 36 male patients and 164 female patients. Other acoustic parameters did not show statistically significant results. (More detailed values are presented in Table 7).

### 3.8. Acoustic Parameter Differences Based on BMI

The BMI category also showed significant associations with voice parameters. Shimmer at the first POM was 13.18% in participants with normal BMI, 15.90% in overweight participants, and 16.72% in obese participants (*p* = 0.001). At the second POM, jitter was 0.46% in normal BMI participants, 0.64% in overweight participants, and 0.72% in obese participants (*p* = 0.003). Similarly, shimmer values were higher among overweight and obese participants across both the second and third POMs. Other acoustic parameters did not show statistically significant results. (More detailed values are presented in Table 8).

### 3.9. Multivariate Analysis of Postoperative Measurements

Multivariate analysis identified significant differences in voice parameters based on the type of surgery at the first POM (Wilks’ Lambda = 0.926, F = 2.680, *p* = 0.023). Gender differences were also significant, with males showing higher variance across postoperative measurements (Wilks’ Lambda = 0.644, F = 18.604, *p* < 0.001 for the first POM). Surgery duration impacted voice parameters at the second and third POMs (*p* = 0.026 and *p* = 0.014, respectively). (More detailed values are presented in Table 8, Table 9, Table 10 and Table 11).

### 3.10. Logistic Regression

Logistic regression indicated that the type of surgery was the most significant predictor of voice disorder development (Exp(B) = 16.533, *p* = 0.001), followed by thyroid volume (Exp(B) = 2.335, *p* = 0.023). The model demonstrated an overall classification accuracy of 82.7%, with Cox and Snell R^2^ of 0.170 and Nagelkerke R^2^ of 0.279. (More detailed values are presented in Table 12).

## 4. Discussion

The significant associations between surgery type, duration, thyroid volume, and BMI with postoperative voice disorders underscore the complex interplay between surgical, anatomical, and patient-related factors that influence voice outcomes after thyroid surgery.

The finding that 82.3% of patients developed postoperative objective voice disorders is consistent with recent studies showing that voice alterations are prevalent even without recurrent laryngeal nerve (RLN) injury. Lee et al. in their study found that negative voice outcomes are present up to 80%. Moreover, Sinagra et al. reported that 87% (40 out of 46 patients) experienced voice changes after thyroidectomy [3]. On the other hand, some authors like Vicente et al. and Iyomasa et al. reported lower values, 46% after total thyroidectomy and 14% of patients after lobectomy by Vicente et al. and 27.8% by Iyomasa et al. [23,24,25].

In our study, the high rate, observed in both lobectomy and total thyroidectomy groups, supports the assertion that structural changes during thyroidectomy, such as strap muscle manipulation, significantly contribute to postoperative dysphonia [8]. Voice disorder incidence was lower among lobectomy patients than among patients who underwent total thyroidectomy, highlighting the impact of surgical complexity on voice outcomes; the same results were confirmed for not only voice disorders but other complications as well following thyroidectomy [5,26,27,28]. Acoustic measurements, including intensity, jitter, and shimmer, revealed significant differences between lobectomy and total thyroidectomy patients, particularly in the first and second postoperative measurements. Moreover, according to the results presented in Table 3, significant differences were observed in voice intensity, as well as in jitter, shimmer, and phonation time during the first postoperative measurement. Three months after surgery (second postoperative measurement) and six months postoperatively (third postoperative measurement), differences in voice intensity, jitter, and shimmer values persisted. All these parameters were poorer in participants who underwent total thyroidectomy compared to those who underwent lobectomy. Patients undergoing total thyroidectomy often exhibit greater deviations in acoustic parameters. Lower intensity and higher jitter and shimmer values in total thyroidectomy patients indicate impaired vocal fold vibration. Lobectomy patients consistently showed better acoustic outcomes across all postoperative measurements. These results are consistent with others in the literature [28].

Our finding that prolonged surgery duration (>90 min) is associated with a higher prevalence of voice disorders corroborates findings in the literature [9,24,29,30]. Surgery duration significantly influences postoperative voice parameters, as shown in Table 6, comparing outcomes between surgeries lasting ≤90 min and >90 min across three postoperative measurements. During the first postoperative measurement, surgeries lasting >90 min resulted in lower intensity (*p* = 0.003), higher jitter (*p* = 0.001), and shorter phonation time (*p* = 0.014). F0 (*p* = 0.780) and shimmer (*p* = 0.090) differences were not statistically significant. In the second postoperative measurement, the trends persisted, with longer surgeries associated with lower intensity (*p* = 0.001), higher jitter (*p* < 0.001), and higher shimmer (*p* < 0.001). Differences in phonation time were no longer significant (*p* = 0.370), while F0 remained unaffected (*p* = 0.028). By the third postoperative measurement, significant differences were observed in jitter (*p* = 0.002) and shimmer (*p* = 0.002), while differences in F0, intensity, and phonation time were not statistically significant (all *p* > 0.05). These findings indicate that longer surgeries lead to more pronounced and prolonged impairments in jitter and shimmer values, while other parameters such as F0, intensity, and phonation time tend to stabilize over time, reflecting partial recovery by six months postoperatively. Vetshev et al. in their study found, using logistic regression models, that surgery duration impacts vocal paresis and paralysis; however, it did not impact persistent postoperative dysphonia [31]. Studies suggest that extended operations increase tissue exposure to intra-operative oedema and stress, potentially affecting the laryngeal framework [32]. Elevated jitter and shimmer values in prolonged surgeries, both indicators of reduced voice quality, further support these findings. Research also shows that longer anaesthesia duration exacerbates laryngeal muscle strain and delays recovery [33]. Our findings that surgery duration influences voice quality, especially in jitter and shimmer, align with studies highlighting the importance of operative time management. Extended surgeries increase intubation-related laryngeal trauma, affecting postoperative phonation time, supporting the notion that shorter surgery duration reduces recovery time [34].

Increased thyroid volume was associated with a higher incidence of voice disorders, paralleling findings by Jain et al. and Vetshev et al. [29,31]. Thyroid volume influences postoperative voice parameters, as detailed in Table 5. During the first postoperative measurement, participants with normal thyroid volume exhibited higher F0 values (*p* = 0.086), suggesting a trend toward better outcomes, although this difference was not statistically significant. Similarly, no significant differences were observed between the two groups in intensity (*p* = 0.128), jitter (*p* = 0.271), shimmer (*p* = 0.771), or phonation time (*p* = 0.192). In the second postoperative measurement, no significant differences were noted across any voice parameters. F0 values were slightly higher in the normal thyroid volume group (*p* = 0.237), but intensity, jitter, shimmer, and phonation time also showed no significant differences (all *p* > 0.05). By the third postoperative measurement, a significant difference emerged in jitter, with participants who had an enlarged thyroid volume exhibiting higher values (*p* = 0.049). Other parameters, including F0, intensity, shimmer, and phonation time, remained statistically insignificant (all *p* > 0.05), indicating stabilization over time. Overall, thyroid volume has a limited impact on most voice parameters, particularly in the early stages of recovery. However, jitter reflects a slower recovery in individuals with an enlarged thyroid volume, emphasizing the need for closer monitoring of this parameter during postoperative rehabilitation. Larger thyroid tumors are more likely to be associated with transient change in voice quality following thyroidectomy despite preservation of recurrent laryngeal nerves [29]. These findings highlight the importance of careful preoperative assessment of thyroid volume as a predictor of postoperative voice outcomes [35].

Park et al. proposed the use of anti-adhesive dressing positioned between the laryngeal unit and the infrahyoid muscles with the overlaying fat, leading to a postoperative voice quality improvement [36].

In our study, the lack of association between age and postoperative voice disorders differs from some previous studies [31,37]. Moreover, Sahli et al. found in their study on multivariate analysis a significant increase in voice or swallowing alterations up to the age of 50 years (5% increased odds per year), after which these changes plateaued [37]. Furthermore, they described that 16% of their patients had voice changes following thyroidectomy (148 from 924 total patients), highlighting that age can play a significant role [37]. Morteza et al., similar to us, did not find statistically significant results when observing age as a factor for developing voice disorders post thyroidectomy. They emphasize that this could be due to relatively small sample size [38]. Although some studies suggest that older patients experience slower recovery, potentially due to reduced tissue elasticity, our study did not observe significant differences. This may be attributable to the age distribution in our cohort or individualized postoperative care practices. Recent studies increasingly question age as important predictor [35,38].

Gender analysis, however, showed strong significance when comparing parameter changes in fundamental frequency and changes in time of phonation across all three POMs to the preoperative measurements. There was no significant findings on whether male or female patients develop more disorders. However, there was a difference in the number of patients of each gender, which most likely contributed to the result (36 male patients and 164 female patients). Gender differences in postoperative voice parameters are detailed in Table 8. During the first postoperative measurement, females exhibited significantly greater changes in F0 compared to males (*p* < 0.001). Phonation time also showed a significant difference, with males exhibiting larger deviations from preoperative values (*p* < 0.001). No significant differences were observed in intensity (*p* = 0.989), jitter (*p* = 0.075), or shimmer (*p* = 0.101). In the second postoperative measurement, similar trends persisted. Females demonstrated greater deviations in F0 compared to males (*p* < 0.001), while males continued to show more pronounced deviations in phonation time (*p* < 0.001). Intensity (*p* = 0.860), jitter (*p* = 0.175), and shimmer (*p* = 0.711) did not differ significantly between genders. By the third postoperative measurement, significant differences were again observed in F0 (*p* < 0.001) and phonation time (*p* < 0.001), with females showing larger deviations in F0 and males showing larger deviations in phonation time. No significant gender differences were noted for intensity (*p* = 0.661), jitter (*p* = 0.049), or shimmer (*p* = 0.867), indicating that these parameters stabilized over time. Overall, gender significantly influences postoperative changes in F0 and phonation time, with females experiencing greater shifts in F0 and males experiencing greater shifts in phonation time across all postoperative measurements. Other authors confirmed the differences but suggested that males suffer from more voice disorders in the early postoperative period [28,35,38].

The association between higher BMI and an increased incidence of voice disorders suggests that excess body weight may exacerbate postoperative complications. BMI is another factor influencing postoperative voice parameters, as detailed in Table 9. During the first postoperative measurement, significant differences were observed in shimmer values, with participants in the normal BMI group exhibiting lower values compared to the overweight and obese groups (*p* = 0.001). No significant differences were found for F0 (*p* = 0.281), intensity (*p* = 0.578), jitter (*p* = 0.176), or phonation time (*p* = 0.429). In the second postoperative measurement, shimmer differences remained significant, with higher values observed in the overweight and obese groups compared to the normal BMI group (*p* = 0.004). Jitter differences also became significant (*p* = 0.003), with higher values in participants with higher BMI. Other parameters, such as F0 (*p* = 0.479), intensity (*p* = 0.317), and phonation time (*p* = 0.280), showed no significant differences. By the third postoperative measurement, shimmer differences persisted, with participants in the normal BMI group showing better outcomes (*p* = 0.009). No significant differences were observed for F0 (*p* = 0.344), intensity (*p* = 0.207), jitter (*p* = 0.060), or phonation time (*p* = 0.152), indicating stabilization of most parameters over time. Overall, BMI significantly impacts shimmer and jitter values, with participants in the normal BMI category demonstrating better voice quality across measurements. These findings align with recent studies identifying BMI as an independent risk factor for postoperative complications, including voice changes [2]. Overweight and obese participants exhibited elevated jitter and shimmer values across all postoperative measurements, suggesting that increased BMI may impede respiratory control or amplify postoperative inflammation. Lee et al. in their study found that patients with higher BMI more often had permanent voice disorders when compared to the ones with temporary voice disorders; however, they did not find statistical significance in overall analysis [32]. Higher BMI was associated with significantly altered acoustic parameters, particularly in jitter and shimmer. However, some authors did not find any correlation between BMI and thyroidectomy complications [39].

To assess the strength of the effects of individual factors on the composite (linear combination) of objective voice quality measures, a multivariate analysis of variance (MANOVA) was conducted for each of the three postoperative measurements. The analysis was based on factors identified as significant in prior analyses. Consequently, the MANOVA results are presented for a slightly smaller sample compared to the total number of participants who developed voice disorders. Given that gender was one of the independent variables tested, changes in voice relative to preoperative measurements were included in the analysis for parameters such as fundamental frequency, voice intensity, and phonation time. This approach allowed for a more nuanced understanding of the impact of surgery on voice changes by accounting for gender-specific variations. According to the results presented in Table 8, within this model, only gender (F = 18.604, *p* < 0.001) and type of surgery (F = 2.680, *p* < 0.05) exhibit significant effects on the levels and changes in voice parameters immediately following thyroidectomy (first measurement). Gender demonstrates the strongest effect, with a partial eta-squared value of 0.356, explaining 35.6% of the variance in objective voice parameters, whereas the type of surgery accounts for 7.4% of the variance. The results presented in Table 9 indicate that during the second postoperative measurement, the situation differs slightly compared to the first measurement. In this case, the duration of surgery (F = 2.615, *p* < 0.05) and gender (F = 2.680, *p* < 0.05) have significant effects on the levels and changes in voice parameters. These two factors can therefore be identified as the most responsible for persistent changes in objective voice measures up to three months after surgery. Similar to the first postoperative measurement, gender exhibits the strongest effect, explaining 49.8% of the variance in objective voice parameters, while the duration of surgery accounts for 7.4%. Finally, in the third postoperative measurement, conducted six months after surgery, the results remain similar to those observed in the second postoperative measurement. Gender and the duration of surgery continue to be the most significant factors influencing substantial changes in objective voice parameters, with gender again playing the more prominent role (Table 10).

The logistic regression model identified surgery type as the most significant predictor of voice disorder development, consistent with studies indicating that more extensive thyroid surgeries carry a greater risk of postoperative dysphonia [24,32,35,40]. Additionally, thyroid volume emerged as a significant predictor, supporting findings that larger glands necessitate more extensive dissection, increasing trauma to vocal structures, similar to our results. Jain et al., in their study on multivariate analysis, found that both weight and volume affected the range of frequency and range of intensity of the voice [23,29]. Our model’s classification accuracy (82.7%) highlights risk factors in post thyroidectomy patients.

Kim et al., in their retrospective study, found using univariate logistic regression analyses that factors related to low-pitched voice were female sex, older age, and low body weight; in addition, multivariate analyses showed that female sex and older age were significantly associated with a negative prognosis for low-pitched voice 1 month after thyroidectomy (odds ratios 0.41 and 1.04, respectively; *p* < 0.001, *N* = 2297) [41].

These findings emphasize the importance of tailored preoperative assessments, considering factors like thyroid volume, BMI, and expected surgery duration. Future research should further examine the mechanistic pathways by which voice disorders arise in post thyroidectomy patients.

Our study’s findings diverge from several points in the literature. Unlike some studies that observed a significant association between age and postoperative voice disorders, particularly with increased odds up to 50 years, we found no statistically significant influence of age on voice outcomes, aligning with results suggesting small sample size limitations but contrasting with the general consensus on age-related recovery differences. Additionally, while some studies reported a prevalence of permanent voice disorders associated with higher BMI, we identified alterations in acoustic parameters like jitter and shimmer in overweight and obese individuals but did not observe overall long-term voice disorder prevalence differences. Furthermore, our study demonstrated prolonged impacts on acoustic parameters like jitter and shimmer linked to extended surgical durations, contrasting with findings that surgery duration impacts vocal paresis and paralysis but not persistent dysphonia. Finally, our results showed no significant gender predisposition to voice disorders, differing from studies that identified female sex and older age as significant predictors of low-pitched voice postoperatively.

## 5. Conclusions

Our study underscores that type and extent of thyroidectomy, thyroid volume, and patient BMI are, most likely, significant predictors of voice changes post-thyroidectomy, with total thyroidectomy patients facing a higher incidence of voice disorders. Acoustic analysis demonstrated pronounced alterations in voice parameters in these cases, highlighting the role of structural changes and laryngeal strain in voice quality degradation. Our findings emphasize the need for meticulous preoperative assessments and enhanced surgical techniques to mitigate voice disorder risks, especially in individuals with large thyroids or higher BMI. Future research should explore interventions and mechanistic insights to minimize these complications and improve postoperative recovery and quality of life for thyroidectomy patients.

## Figures and Tables

**Table 1:** Examples of similar studies.

Study Title	Sample Size	Assessment Tools	Type
“Time-Related Voice and Swallowing Symptoms in Patients After Thyroidectomy Without Clinical Evidence of Recurrent Laryngeal Nerve Injury”	56 patients	Subjective assessments over 6 months	Prospective study
“Impact of Thyroid Surgery on Voice: A Prospective Study in Patients Without Recurrent Laryngeal Nerve Injury”	20 patients	Voice Impairment Score (VIS), Multi-Dimensional Voice Program (MDVP)	Prospective study
“Voice Outcomes After Thyroidectomy Without Superior and Recurrent Laryngeal Nerve Injury: A Prospective Analysis”	39 patients	Voice Symptom Scale (VoiSS), GRBAS scale	Prospective study
“Long-Term Voice Quality Outcomes After Total Thyroidectomy: A Prospective Study”	203 patients	Voice Handicap Index (VHI)	Prospective study
“A Multivariate Analysis of Objective Voice Changes After Thyroidectomy Without Laryngeal Nerve Injury”	36 patients	Multi-Dimensional Voice Program (MDVP)	Prospective study
“Voice Changes Without Laryngeal Nerve Alterations After Thyroidectomy: The Need For Prospective Trials—A Review Study”			Literature review
“Effects on Voice Quality of Thyroidectomy: A Qualitative and Quantitative Study Using Voice Maps”	57 patients	Comparative voice maps, pre- and post-intervention, of six acoustic and electroglottographic (EGG) metrics	Retrospective study

**Table 2:** Surgical characteristics and socio-demographic data.

		Total	Type of Surgery	Type of Surgery
		Total	Lobectomy	Total Thyroidectomy
Total		243	141	102
Voice Disorder	No Voice Disorder	43	41	2
Voice Disorder	Developed Voice Disorder	200	100	100
Surgery Duration	Up to 60 min.	58	56	2
Surgery Duration	61 to 90 min.	84	65	19
Surgery Duration	91 to 120 min.	46	17	29
Surgery Duration	Over 120 min.	55	3	52
Thyroid Volume	Normal	118	68	50
Thyroid Volume	Enlarged	125	73	52
Gender	Male	42	23	19
Gender	Female	201	118	83
Age	20 to 39 years	59	31	28
Age	40 to 59 years	138	80	58
Age	60 to 79 years	46	30	16
BMI	Normal	39	31	8
BMI	Overweight	111	62	49
BMI	Obese	93	48	45
Alcohol Consumption	Less than once a week	157	85	72
Alcohol Consumption	Once a week or more	86	56	30
Smoking Status	Non-smokers	129	73	56
Smoking Status	Smokers	114	68	46

**Table 3:** Development of voice disorders relative to surgery type, socio-demographic characteristics, and lifestyle factors.

		Voice Disorder	Voice Disorder	Total	Χ2	*p*
		No Voice Disorder	Voice Disorder	Total	Χ2	*p*
*N*		43	200	243		
Type of Surgery	Lobectomy	95.3%	50.0%	58.0%	29.88	0.000
Type of Surgery	Total Thyroidectomy	4.7%	50.0%	42.0%	29.88	0.000
Surgery Duration	<90 min.	86.0%	52.5%	58.4%	16.40	0.000
Surgery Duration	>90 min.	14.0%	47.5%	41.6%	16.40	0.000
Thyroid Volume	Normal	62.8%	45.5%	48.6%	4.24	0.045
Thyroid Volume	Enlarged	37.2%	54.5%	51.4%	4.24	0.045
Gender	Male	14.0%	18.0%	17.3%	0.41	0.659
Gender	Female	86.0%	82.0%	82.7%	0.41	0.659
Age	20 to 39 years	23.3%	24.5%	24.3%	0.14	0.932
Age	40 to 59 years	55.8%	57.0%	56.8%	0.14	0.932
Age	60 to 79 years	20.9%	18.5%	18.9%	0.14	0.932
BMI	Normal	30,2%	13.5%	16.5%	8.97	0.011
BMI	Overweight	46.5%	45.5%	45.7%	8.97	0.011
BMI	Obese	23.3%	41.0%	37.9%	8.97	0.011
Smoking Status	Non-smokers	53.5%	53.0%	53.1%	0.00	0.545
Smoking Status	Smokers	46.5%	47.0%	46.9%	0.00	0.545
Alcohol Consumption	Less than once a week	53.5%	73.0%	69.5%	2.83	0.114
Alcohol Consumption	Once a week or more	46.5%	27.0%	30.5%	2.83	0.114

**Table 4:** Differences in average values of objective voice parameters based on type of surgery.

Type of Surgery	*N*	Mean	STD. Deviation	*p*-Value
F0 (1st postoperative measurement)				
Lobectomy	100	170.30	32.536	0.204
Total Thyroidectomy	100	171.12	31.845	
Intensity (1st postoperative measurement)				
Lobectomy	100	69.72	9.443	<0.001
Total Thyroidectomy	100	66.03	10.536	
Jitter (1st postoperative measurement)				
Lobectomy	100	0.94	0.382	<0.001
Total Thyroidectomy	100	1.10	0.550	
Shimmer (1st postoperative measurement)				
Lobectomy	100	15.08	3.652	0.006
Total Thyroidectomy	100	16.65	4.699	
Phonation Time (1st postoperative measurement)				
Lobectomy	100	17.48	3.645	<0.001
Total Thyroidectomy	100	14.83	3.613	
F0 (2nd postoperative measurement)				
Lobectomy	100	178.12	27.71	0.440
Total Thyroidectomy	100	175.41	28.76	
Intensity (2nd postoperative measurement)				
Lobectomy	100	70.45	7.416	0.001
Total Thyroidectomy	100	67.84	5.216	
Jitter (2nd postoperative measurement)				
Lobectomy	100	0.57	0.384	<0.001
Total Thyroidectomy	100	0.73	0.339	
Shimmer (2nd postoperative measurement)				
Lobectomy	100	8.10	5.730	0.001
Total Thyroidectomy	100	10.60	4.943	
Phonation Time (2nd postoperative measurement)				
Lobectomy	100	18.54	3.128	0.224
Total Thyroidectomy	100	19.21	2.880	
F0 (3rd postoperative measurement)				
Lobectomy	100	178.26	27.531	0.560
Total Thyroidectomy	100	177.70	25.827	
Intensity (3rd postoperative measurement)				
Lobectomy	100	70.58	4.562	0.001
Total Thyroidectomy	100	68.66	6.107	
Jitter (3rd postoperative measurement)				
Lobectomy	100	0.49	0.317	0.046
Total Thyroidectomy	100	0.59	0.372	
Shimmer (3rd postoperative measurement)				
Lobectomy	100	6.16	4.699	0.041
Total Thyroidectomy	100	7.62	4.808	
Phonation Time (3rd postoperative measurement)				
Lobectomy	100	18.66	3.026	0.258
Total Thyroidectomy	100	19.27	2.849	

**Table 5:** Differences in average values of objective voice parameters based on surgery Duration.

Duration of Surgery	*N*	Mean	STD. Deviation	*p*-Value
F0 (1st postoperative measurement)				
≤90 min	105	171.41	34.010	0.780
>90 min	95	169.94	30.040	
Intensity (1st postoperative measurement)				
≤90 min	105	69.63	9.809	0.003
>90 min	95	65.93	10.213	
Jitter (1st postoperative measurement)				
≤90 min	105	0.94	0.370	0.001
>90 min	95	1.11	0.565	
Shimmer (1st postoperative measurement)				
≤90 min	105	15.43	3.702	0.090
>90 min	95	16.35	4.796	
Phonation Time (1st postoperative measurement)				
≤90 min	105	16.84	3.826	0.014
>90 min	95	15.40	3.756	
F0 (2nd postoperative measurement)				
≤90 min	105	180.80	26.678	0.028
>90 min	95	172.31	29.292	
Intensity (2nd postoperative measurement)				
≤90 min	105	70.64	6.746	0.001
>90 min	95	67.49	5.883	
Jitter (2nd postoperative measurement)				
≤90 min	105	0.54	0.346	<0.001
>90 min	95	0.77	0.361	
Shimmer (2nd postoperative measurement)				
≤90 min	105	7.90	5.516	<0.001
>90 min	95	10.95	5.000	
Phonation Time (2nd postoperative measurement)				
≤90 min	105	18.63	3.136	0.370
>90 min	95	19.15	2.873	
F0 (3rd postoperative measurement)				
≤90 min	105	181.02	26.405	0.057
>90 min	95	174.62	26.604	
Intensity (3rd postoperative measurement)				
≤90 min	105	69.53	4.483	0.341
>90 min	95	69.72	6.396	
Jitter (3rd postoperative measurement)				
≤90 min	105	0.46	0.280	0.002
>90 min	95	0.63	0.393	
Shimmer (3rd postoperative measurement)				
≤90 min	105	5.93	4.584	0.002
>90 min	95	7.95	4.828	
Phonation Time (3rd postoperative measurement)				
≤90 min	105	18.74	3.035	0.415
>90 min	95	19.21	2.843	

**Table 6:** Differences in average values of objective voice parameters based on thyroid volume.

Thyroid Volume	*N*	Mean	Std. Deviation	*p*-Value
F0 (1st postoperative measurement)				
Normal	91	175.90	33.028	0.086
Enlarged	109	166.38	30.816	
Intensity (1st postoperative measurement)				
Normal	91	67.44	11.431	0.128
Enlarged	109	68.24	8.978	
Jitter (1st postoperative measurement)				
Normal	91	1.02	0.419	0.271
Enlarged	109	1.02	0.526	
Shimmer (1st postoperative measurement)				
Normal	91	16.02	4.519	0.771
Enlarged	109	15.74	4.068	
Phonation Time (1st postoperative measurement)				
Normal	91	15.74	3.590	0.192
Enlarged	109	16.50	4.047	
F0 (2nd postoperative measurement)				
Normal	91	178.74	29.117	0.237
Enlarged	109	175.12	27.439	
Intensity (2nd postoperative measurement)				
Normal	91	69.03	6.299	0.784
Enlarged	109	69.24	6.740	
Jitter (2nd postoperative measurement)				
Normal	91	0.64	0.401	0.393
Enlarged	109	0.66	0.344	
Shimmer (2nd postoperative measurement)				
Normal	91	9.35	5.863	0.740
Enlarged	109	9.35	5.169	
Phonation Time (2nd postoperative measurement)				
Normal	91	18.95	2.987	0.816
Enlarged	109	18.82	3.056	
F0 (3rd postoperative measurement)				
Normal	91	181.80	26.105	0.052
Enlarged	109	174.79	26.756	
Intensity (3rd postoperative measurement)				
Normal	91	69.77	0.371	0.446
Enlarged	109	69.50	0.328	
Jitter (3rd postoperative measurement)				
Normal	91	0.51	0.371	0.049
Enlarged	109	0.57	0.328	
Shimmer (3rd postoperative measurement)				
Normal	91	6.81	5.350	0.177
Enlarged	109	6.96	4.308	
Phonation Time (3rd postoperative measurement)				
Normal	91	18.99	2.968	0.956
Enlarged	109	18.95	2.943	

**Table 7:** Differences in average deviations of objective voice parameters relative to preoperative measurements based on gender.

Gender	*N*	Mean	Std. Deviation	*p*-Value
Change in F0 (1st postoperative measurement)				
Male	36	0.56	32.192	<0.001
Female	164	22.95	28.905	
Change in Intensity (1st postoperative measurement)				
Male	36	2.25	10.263	0.989
Female	164	2.65	10.423	
Jitter (1st postoperative measurement)				
Male	36	1.04	0.354	0.075
Female	164	1.02	0.503	
Shimmer (1st postoperative measurement)				
Male	36	16.71	3.676	0.101
Female	164	15.68	4.379	
Change in Phonation Time (1st postoperative measurement)				
Male	36	11.42	4.837	<0.001
Female	164	2.88	4.612	
Change in F0 (2nd postoperative measurement)				
Male	36	−6.14	28.663	<0.001
Female	164	14.33	25.655	
Change in Intensity (2nd postoperative measurement)				
Male	36	1.86	4.975	0.860
Female	164	1.19	7.073	
Jitter (2nd postoperative measurement)				
Male	36	0.60	0.400	0.175
Female	164	0.66	0.364	
Shimmer (2nd postoperative measurement)				
Male	36	8.90	5.021	0.711
Female	164	9.45	5.587	
Change in Phonation Time (2nd postoperative measurement)				
Male	36	8.31	3.446	<0.001
Female	164	0.24	4.431	
Change in F0 (3rd postoperative measurement)				
Male	36	−5.36	28.210	<0.001
Female	164	11.55	22.633	
Change in Intensity (3rd postoperative measurement)				
Male	36	2.00	5.918	0.661
Female	164	0.58	5.894	
Jitter (3rd postoperative measurement)				
Male	36	0.51	0.371	0.049
Female	164	0.57	0.328	
Shimmer (3rd postoperative measurement)				
Male	36	6.52	4.237	0.867
Female	164	6.97	4.921	
Change in Phonation Time (3rd postoperative measurement)				
Male	36	8.31	3.454	<0.001
Female	164	0.13	4.367	

**Table 8:** Differences in average values of objective voice parameters based on body mass index (BMI).

BMI Category	*N*	Mean	Std. Deviation	*p*-Value
F0 (1st postoperative measurement)				
Normal	27	175.96	21.945	0.281
Overweight	91	170.18	33.325	
Obese	82	169.57	33.680	
Intensity (1st postoperative measurement)				
Normal	27	68.15	8.939	0.578
Overweight	91	67.43	10.970	
Obese	82	68.28	9.659	
Jitter (1st postoperative measurement)				
Normal	27	0.87	0.173	0.176
Overweight	91	1.06	0.565	
Obese	82	1.03	0.436	
Shimmer (1st postoperative measurement)				
Normal	27	13.18	3.234	0.001
Overweight	91	15.90	4.245	
Obese	82	16.72	4.276	
Phonation Time (1st postoperative measurement)				
Normal	27	19.52	3.056	0.429
Overweight	91	18.80	2.758	
Obese	82	18.74	3.280	
F0 (2nd postoperative measurement)				
Normal	27	179.70	27.480	0.479
Overweight	91	174.37	27.713	
Obese	82	178.45	29.087	
Intensity (2nd postoperative measurement)				
Normal	27	70.22	7.562	0.317
Overweight	91	68.35	6.954	
Obese	82	69.67	5.587	
Jitter (2nd postoperative measurement)				
Normal	27	0.46	0.209	0.003
Overweight	91	0.64	0.375	
Obese	82	0.72	0.386	
Shimmer (2nd postoperative measurement)				
Normal	27	5.84	3.212	0.004
Overweight	91	9.46	5.369	
Obese	82	10.39	5.769	
Phonation Time (2nd postoperative measurement)				
Normal	27	19.52	3.056	0.280
Overweight	91	18.80	2.758	
Obese	82	18.74	3.280	
F0 (3rd postoperative measurement)				
Normal	27	183.00	22.424	0.344
Overweight	91	174.74	27.715	
Obese	82	179.93	26.512	
Intensity (3rd postoperative measurement)				
Normal	27	70.19	1.798	0.207
Overweight	91	68.99	5.355	
Obese	82	70.13	6.291	
Jitter (3rd postoperative measurement)				
Normal	27	0.38	0.152	0.060
Overweight	91	0.58	0.385	
Obese	82	0.55	0.340	
Shimmer (3rd postoperative measurement)				
Normal	27	4.06	0.899	0.009
Overweight	91	7.65	4.939	
Obese	82	6.97	5.086	
Phonation Time (3rd postoperative measurement)				
Normal	27	19.52	3.056	0.152
Overweight	91	18.80	2.758	
Obese	82	18.74	3.280	

**Table 9:** Multivariate analysis of postoperative measurement–POM 1.

		*N*	Wilks’ Lambda	F	Significance	Partial Eta Squared	Observed Power
Type of Surgery	Lobectomy	92	0.926	2.680	0.023	0.074	0.805
Type of Surgery	Total Thyroidectomy	87	0.926	2.680	0.023	0.074	0.805
Thyroid Volume	Normal	80	0.987	0.434	0.825	0.013	0.163
Thyroid Volume	Enlarged	99	0.987	0.434	0.825	0.013	0.163
Surgery Duration	<90 min	95	0.959	1.439	0.213	0.041	0.498
Surgery Duration	>90 min	84	0.959	1.439	0.213	0.041	0.498
Gender	Male	32	0.644	18.604	0.000	0.356	1.000
Gender	Female	147	0.644	18.604	0.000	0.356	1.000
BMI Category	Normal	25	0.911	1.596	0.106	0.045	0.778
BMI Category	Overweight	81	0.911	1.596	0.106	0.045	0.778
BMI Category	Obese	73	0.911	1.596	0.106	0.045	0.778

**Table 10:** Multivariate analysis of postoperative measurement–POM 2.

		*N*	Wilks’ Lambda	F	Significance	Partial Eta Squared	Noncent. Parameter	Observed Powerd
Type of Surgery	Lobectomy	89	0.948	1.812	0.113	0.052	9.061	0.610
Type of Surgery	Total Thyroidectomy	86	0.948	1.812	0.113	0.052	9.061	0.610
Thyroid Volume	Normal	79	0.973	0.920	0.470	0.027	4.598	0.323
Thyroid Volume	Enlarged	96	0.973	0.920	0.470	0.027	4.598	0.323
Surgery Duration	<90 min.	96	0.926	2.615	0.026	0.074	13.074	0.793
Surgery Duration	>90 min.	79	0.926	2.615	0.026	0.074	13.074	0.793
Gender	Male	33	0.502	32.475	0.000	0.498	162.373	1.000
Gender	Female	142	0.502	32.475	0.000	0.498	162.373	1.000
BMI Category	Normal	25	0.906	1.666	0.087	0.048	16.661	0.799
BMI Category	Overweight	78	0.906	1.666	0.087	0.048	16.661	0.799
BMI Category	Obese	72	0.906	1.666	0.087	0.048	16.661	0.799

**Table 11:** Multivariate analysis of postoperative measurement–POM 3.

		*N*	Wilks’ Lambda	F	Significance	Partial Eta Squared	Noncent. Parameter	Observed Powerd
Type of Surgery	Lobectomy	91	0.960	1.338	0.251	0.040	6.691	0.465
Type of Surgery	Total Thyroidectomy	79	0.960	1.338	0.251	0.040	6.691	0.465
Thyroid Volume	Normal	75	0.956	1.473	0.201	0.044	7.367	0.508
Thyroid Volume	Enlarged	95	0.956	1.473	0.201	0.044	7.367	0.508
Surgery Duration	<90 min.	95	0.915	2.961	0.014	0.085	14.803	0.847
Surgery Duration	>90 min.	75	0.915	2.961	0.014	0.085	14.803	0.847
Gender	Male	24	0.557	25.289	0.000	0.443	126.444	1.000
Gender	Female	146	0.557	25.289	0.000	0.443	126.444	1.000
BMI Category	Normal	27	0.934	1.106	0.357	0.034	11.063	0.580
BMI Category	Overweight	73	0.934	1.106	0.357	0.034	11.063	0.580
BMI Category	Obese	70	0.934	1.106	0.357	0.034	11.063	0.580

**Table 12:** Logistic Regression.

	B	Wald	Significance	Exp(B)	Cox and Snell R Square	Nagelkerke R Square	Overall % of Correctly Classified Cases
Type of Surgery	2.805	11.200	0.001	16.533	0.170	0.279	82.7
Surgery Duration	0.003	0.157	0.692	1.003	0.170	0.279	82.7
Thyroid Volume	0.848	5.137	0.023	2.335	0.170	0.279	82.7
BMI	0.083	2.624	0.105	1.087	0.170	0.279	82.7

## Data Availability

The original contributions presented in this study are included in the article. Further inquiries can be directed to the corresponding author.
